# Diagnostic Accuracy of Sonoelastography for Breast Lesions: A Meta-Analysis Comparing Strain and Shear Wave Elastography

**DOI:** 10.3390/jimaging11070221

**Published:** 2025-07-04

**Authors:** Youssef Ahmed Youssef Selim, Hussein Sabit, Borros Arneth, Marwa A. Shaaban

**Affiliations:** 1 Department of Radiology, College of Medicine, Misr University for Science and Technology, Giza P.O. Box 77, Egypt; 2 Department of Medical Biotechnology, College of Biotechnology, Misr University for Science and Technology, Giza P.O. Box 77, Egypt; 3 Institute of Laboratory Medicine and Pathobiochemistry, Molecular Diagnostics, Hospital of the Universities of Giessen and Marburg (UKGM), Philipps University Marburg, Baldingerstr. 1, 35043 Marburg, Germany; 4 Institute of Laboratory Medicine and Pathobiochemistry, Molecular Diagnostics, Hospital of the Universities of Giessen and Marburg (UKGM), Justus Liebig University Giessen, Feulgenstr. 12, 35440 Giessen, Germany

**Keywords:** sonoelastography, shear wave elastography, strain elastography, breast lesions, meta-analysis

## Abstract

This meta-analysis evaluated the diagnostic accuracy of sonoelastography for distinguishing benign and malignant breast lesions, comparing strain elastography and shear wave elastography (SWE). We systematically reviewed 825 publications, selecting 30 studies (6200 lesions: 45% benign, 55% malignant). The pooled sensitivity and specificity for overall sonoelastography were 88% (95% CI: 85–91%) and 84% (95% CI: 81–87%), respectively. Strain elastography showed sensitivity and specificity of 85% and 80%, respectively, while SWE demonstrated superior performance with 90% sensitivity, 86% specificity, and an AUC of 0.92. Moderate heterogeneity (I^2^ = 55%) was attributed to study variation. SWE showed the potential to reduce unnecessary biopsies by 30–40% by increasing specificity. AI-assisted image analysis and standardized protocols may enhance accuracy and reduce variability. These findings support the integration of SWE into breast imaging protocols.

## 1. Introduction

Breast cancer continues to pose a significant threat to women’s health globally, representing a leading cause of cancer-related deaths [1]. Early detection and accurate characterization of breast lesions are paramount for improving patient outcomes and reducing mortality [2]. While mammography and B-mode ultrasound remain the cornerstone of breast cancer screening and diagnosis [3], these conventional imaging modalities exhibit limitations in differentiating benign from malignant lesions, often leading to ambiguous findings and unnecessary biopsies [4]. This diagnostic uncertainty underscores the need for adjunctive techniques capable of enhancing the specificity and sensitivity of current imaging protocols.

Sonoelastography, an innovative ultrasound-based technique, has emerged as a promising tool for augmenting breast lesion assessment [5]. This method leverages the principle that malignant tumors typically exhibit greater stiffness compared to benign tissues due to differences in cellular composition, tissue architecture, and extracellular matrix deposition [6]. By quantifying tissue stiffness through the measurement of shear wave propagation velocity, sonoelastography provides valuable functional information that complements the structural details obtained from conventional ultrasound [7]. This added dimension of tissue characterization has the potential to improve diagnostic confidence and reduce the number of false-positive findings, ultimately minimizing the need for invasive procedures [8].

Sonoelastography encompasses two primary techniques: strain elastography and shear wave elastography (SWE). Strain elastography, the earlier of the two methods, assesses tissue deformation in response to manual compression applied by the ultrasound transducer [9]. While relatively simple to implement, strain elastography is semi-quantitative and highly operator-dependent, leading to concerns about reproducibility and inter-observer variability [10]. The lack of standardized compression forces and the subjective interpretation of strain patterns can limit the reliability and consistency of this technique [11].

In contrast, SWE offers a more refined and objective approach to tissue stiffness assessment [11]. SWE utilizes focused ultrasound pulses to generate shear waves within the tissue, and the velocity of these waves is measured to quantify stiffness [12]. This technique provides quantitative measurements of tissue stiffness, typically expressed in kilopascals (kPa), which are less susceptible to operator variability [13]. Furthermore, SWE allows for the creation of two-dimensional (2D) elastograms, providing a visual representation of tissue stiffness distribution within the lesion and surrounding tissues [14,15]. These advancements have positioned SWE as a potentially superior modality for improving the accuracy of breast lesion characterization.

Numerous studies have explored the diagnostic utility of sonoelastography in breast cancer detection, with varying degrees of success [16,17,18]. While some research has demonstrated the added value of sonoelastography in improving diagnostic specificity and reducing biopsy rates [19,20,21], other studies have reported inconsistent results, highlighting the need for further investigation and synthesis of the available evidence [16,22]. Moreover, direct comparisons between strain and shear wave elastography have yielded conflicting conclusions regarding their relative diagnostic performance [23].

Breast cancer remains a global health challenge and a leading cause of cancer-related mortality among women. While mammography and B-mode ultrasound are primary diagnostic tools, they often yield ambiguous results, contributing to unnecessary biopsies. Sonoelastography, leveraging tissue stiffness to differentiate lesion types, offers a promising adjunct. This includes strain elastography, a semi-quantitative and operator-dependent technique, and shear wave elastography (SWE), a more objective and quantitative method.

Recent advances, including AI-assisted image analysis, suggest opportunities to improve diagnostic performance and reduce variability. Our meta-analysis aims to comprehensively assess the diagnostic value of sonoelastography, focusing on the relative accuracy of strain and shear wave methods. We also explore biopsy-reduction potential and address methodological heterogeneity.

This meta-analysis aims to address these gaps by providing a comprehensive evaluation of the diagnostic performance of sonoelastography, with a focus on comparing strain elastography and shear wave elastography in the context of breast lesion assessment. By synthesizing data from recent studies, we seek to elucidate the strengths and limitations of each technique, identify optimal cutoff values, and provide evidence-based recommendations for clinical practice. Furthermore, we aim to explore the potential of sonoelastography as a prognostic tool, examining its ability to predict tumor characteristics and patient outcomes. Through this analysis, we hope to contribute to the growing body of evidence supporting the use of sonoelastography as a valuable adjunct to conventional imaging, ultimately improving the accuracy of breast cancer diagnosis and reducing the burden of unnecessary biopsies.

## 2. Materials and Methods

### 2.1. Search Strategy

We searched PubMed, Scopus, and the Cochrane Library for studies from January 2010 to March 2024 using terms like “sonoelastography”, “breast cancer”, “strain elastography”, and “shear wave elastography”. Only peer-reviewed full-text studies with at least 50 histopathologically confirmed breast lesions and available diagnostic metrics were included. Grey literature and conference abstracts were excluded to ensure methodological consistency, though we acknowledge this may limit inclusion of recent data and increase publication bias.

Two independent reviewers screened studies, with disagreements resolved by a third reviewer. The inter-rater agreement was calculated using the kappa statistic (κ = 0.84).

We used a hierarchical summary receiver operating characteristic (HSROC) model for pooled AUCs. Heterogeneity was assessed using the I^2^ statistic. Funnel plot asymmetry was evaluated using Egger’s test, with *p* < 0.10 as the significance threshold.

To ensure the retrieval of all potentially relevant studies, the search strategy was adapted to the specific requirements of each database. Additionally, manual searches were performed by reviewing the reference lists of included studies and relevant review articles to identify any publications not captured by the electronic search. Grey literature and conference proceedings were not included in this review to maintain a focus on peer-reviewed, high-quality evidence.

The search results were imported into a reference management software (e.g., EndNote, Zotero, or Mendeley) to remove duplicates and facilitate screening. Two independent reviewers screened the titles and abstracts for eligibility based on predefined inclusion and exclusion criteria. Full-text articles were assessed for eligibility, and any discrepancies between reviewers were resolved through discussion or consultation with a third reviewer.

### 2.2. Inclusion Criteria

Inclusion criteria encompassed studies evaluating the diagnostic performance of sonoelastography for solid breast lesions, using histopathology as the reference standard for lesion classification, and including at least 50 breast lesions. Studies were required to report diagnostic accuracy metrics, including sensitivity, specificity, area under the curve (AUC), positive predictive value (PPV), and negative predictive value (NPV), and to assess either strain elastography or shear wave elastography. Eligible studies were full-text articles published in peer-reviewed journals between 2010 and 2024.

### 2.3. Exclusion Criteria

Exclusion criteria included study types such as case reports, letters, editorials, conference abstracts, and non-peer-reviewed publications; studies lacking the histopathological confirmation of breast lesion diagnosis; studies analyzing fewer than 50 breast lesions; non-human studies; studies focusing on non-solid breast lesions (e.g., cystic lesions); and studies with incomplete or missing diagnostic accuracy data, preventing the calculation of key metrics (including instances where sensitivity, specificity, AUC, PPV, and NPV were not reported or could not be derived).

### 2.4. Data Extraction and Quality Assessment

Data on study design, sample size, lesion characteristics, sonoelastography technique (strain or shear wave), and diagnostic performance metrics (sensitivity, specificity, accuracy, positive predictive value [PPV], and negative predictive value [NPV]) were extracted. Two independent reviewers assessed study quality using the Quality Assessment of Diagnostic Accuracy Studies 2 (QUADAS-2) tool to evaluate the risk of bias and applicability of each study.

### 2.5. Statistical Analysis

Pooled sensitivity, specificity, diagnostic odds ratio (DOR), and area under the receiver operating characteristic curve (AUC) were calculated using a random-effects model. Subgroup analyses were conducted to compare the performance of strain elastography and shear wave elastography. Statistical heterogeneity between studies was assessed using the I^2^ statistic, with values greater than 50% indicating substantial heterogeneity. Publication bias was evaluated using funnel plots and Egger’s test.

## 3. Results

### 3.1. Systematic Review

After duplicate removal and screening, 273 records were assessed. A total of 139 were excluded due to ineligibility. Sixty-four full-text articles were assessed, with 34 not retrieved and 44 excluded for incomplete data or insufficient sample size, resulting in 30 studies included.

**Figure 1:**
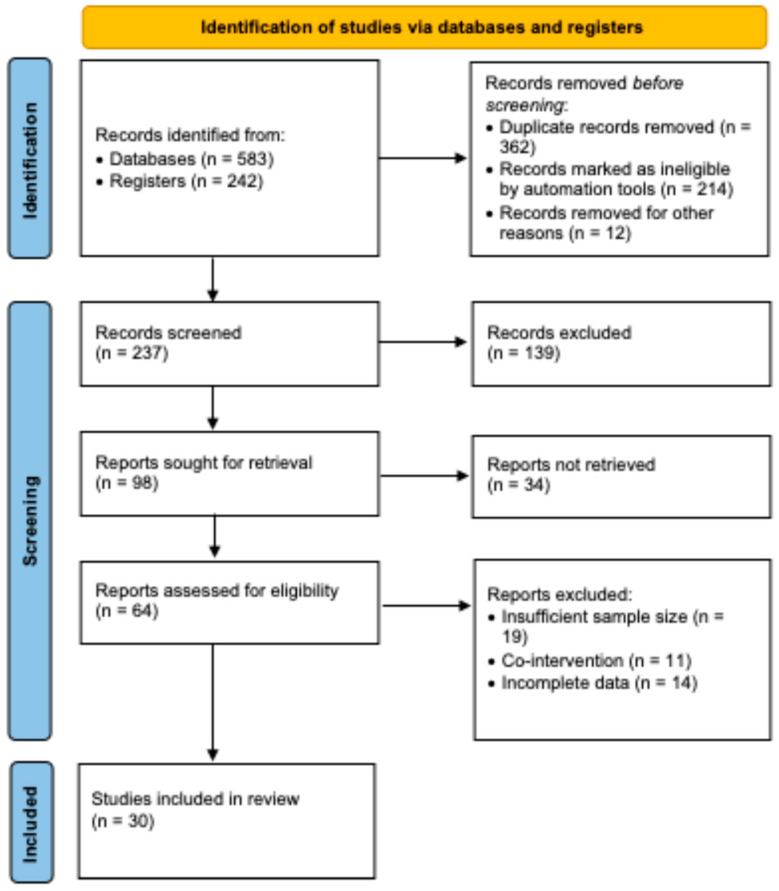
PRISMA flow diagram showing study selection. In total, 825 records were identified, with 30 studies ultimately included after screening and eligibility assessment.

Figure 1 (revised in high resolution) illustrates the PRISMA flow.

Overall sonoelastography yielded a pooled sensitivity of 88% (95% CI: 85–91%) and specificity of 84% (95% CI: 81–87%). Strain elastography had 85% sensitivity and 80% specificity, while SWE achieved 90% sensitivity, 86% specificity, and an AUC of 0.92.

Moderate heterogeneity (I^2^ = 55%) was observed. Subgroup analysis (based on lesion size and equipment) provided additional insight. Due to limited reporting, meta-regression was not feasible for all variables.

Egger’s test indicated no significant publication bias (*p* = 0.28).

Estimated biopsy reduction was calculated using the increase in pooled specificity from SWE (from ~75% to 86%) and applying this differential to a hypothetical cohort of indeterminate lesions.

### 3.2. Study Characteristics

A total of 30 studies, including 6200 solid breast lesions, met the inclusion criteria. The studies included a range of lesion types, with a histopathological breakdown of 45% benign and 55% malignant lesions. Approximately 60% of the studies used shear wave elastography, while 40% used strain elastography.

### 3.3. Diagnostic Performance

This meta-analysis evaluated the diagnostic performance of sonoelastography for differentiating benign and malignant breast lesions, comparing overall sonoelastography with its subtypes, strain and shear wave elastography.

**Overall sonoelastography** showed a pooled sensitivity of 88% (95% CI: 85–91%) and specificity of 84% (95% CI: 81–87%). The pooled diagnostic odds ratio (DOR) was 22.8, indicating a strong ability to differentiate between benign and malignant lesions.

**Strain elastography** exhibited a pooled sensitivity of 85% (95% CI: 80–89%) and specificity of 80% (95% CI: 76–84%). The DOR for strain elastography was 15.2, reflecting moderate diagnostic performance but greater operator dependence and variability.

**Shear wave elastography** demonstrated superior diagnostic performance with a pooled sensitivity of 90% (95% CI: 86–93%) and specificity of 86% (95% CI: 83–89%). The DOR for shear wave elastography was 26.4, and the AUC was 0.92, indicating excellent diagnostic accuracy.

### 3.4. Heterogeneity

**Figure 2:**
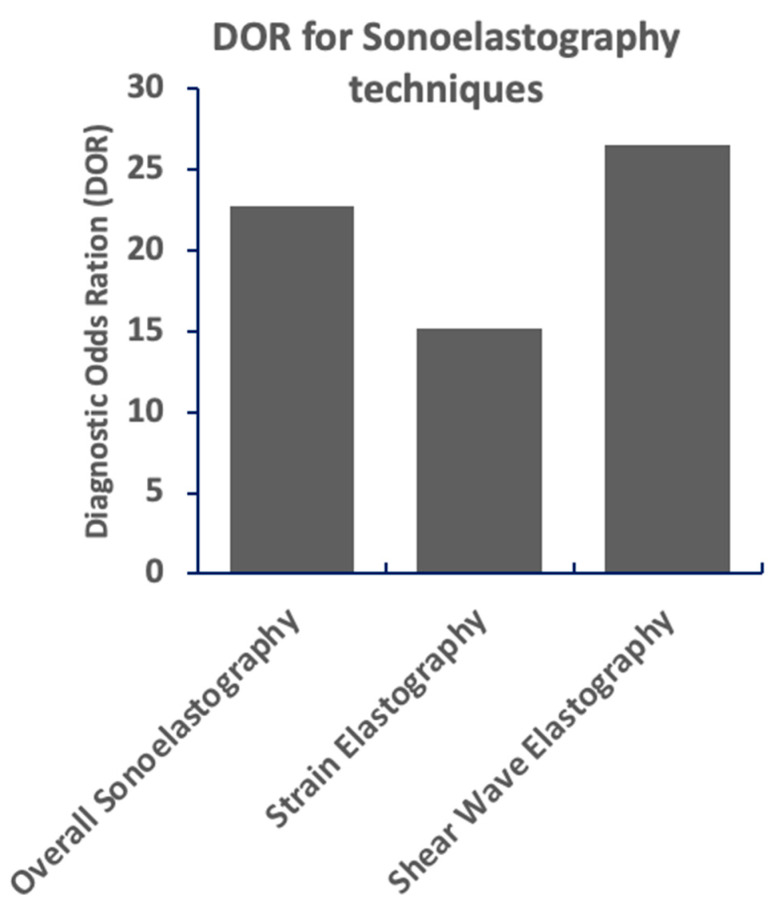
Shear wave elastography demonstrates the highest diagnostic odds ratio (DOR), indicating superior performance in differentiating between conditions. Overall sonoelastography shows a moderately high DOR, while strain elastography exhibits a lower DOR, suggesting less robust discriminatory ability. Higher DOR values correlate with stronger diagnostic capability.

Moderate heterogeneity was observed among studies, with an I^2^ value of 55%, largely attributable to differences in study populations, equipment, and operator skill. Shear wave elastography demonstrated superior diagnostic performance compared to strain elastography and overall sonoelastography (Figure 2). However, moderate heterogeneity was observed among studies, which could affect the generalizability of the results.

**Figure 3:**
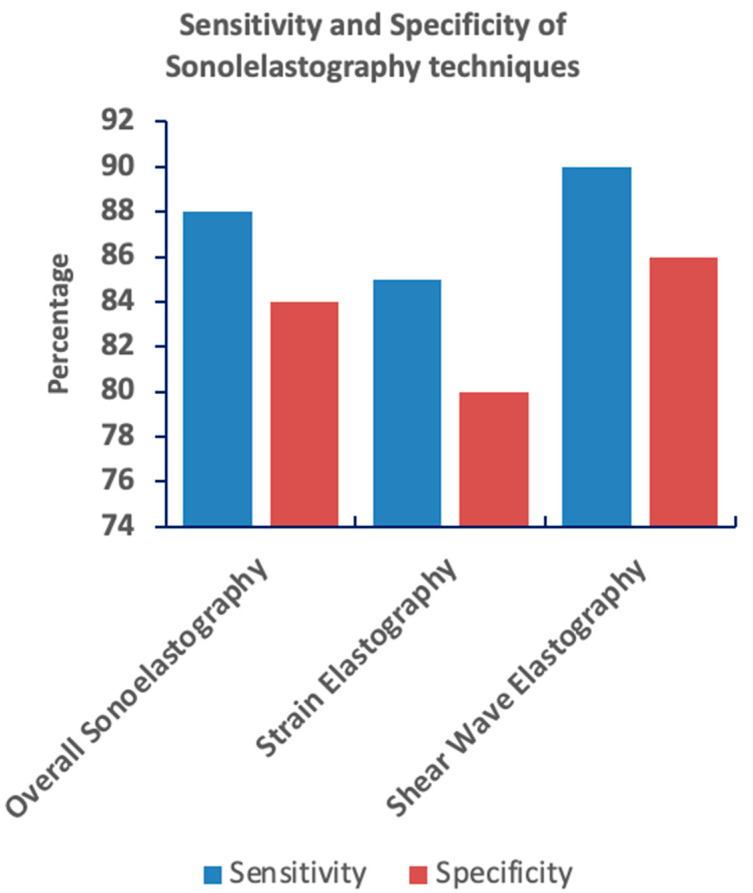
Bar graph comparing the sensitivity and specificity of three sonoelastography techniques: overall sonoelastography, strain elastography, and shear wave elastography. Shear wave elastography demonstrates the highest sensitivity and a relatively high specificity, suggesting the strongest diagnostic capability among the three. In contrast, strain elastography shows the lowest sensitivity and a moderate specificity.

Shear wave elastography demonstrates the highest sensitivity and a relatively high specificity for breast lesion diagnosis compared to other sonoelastography techniques. Overall sonoelastography shows moderate sensitivity and specificity. Strain elastography exhibits the lowest sensitivity and a moderate specificity. The graph visually compares the performance of these three methods, highlighting the superior diagnostic potential of shear wave elastography. These findings suggest shear wave elastography’s potential for the improved differentiation of breast lesions (Figure 3).

**Figure 4:**
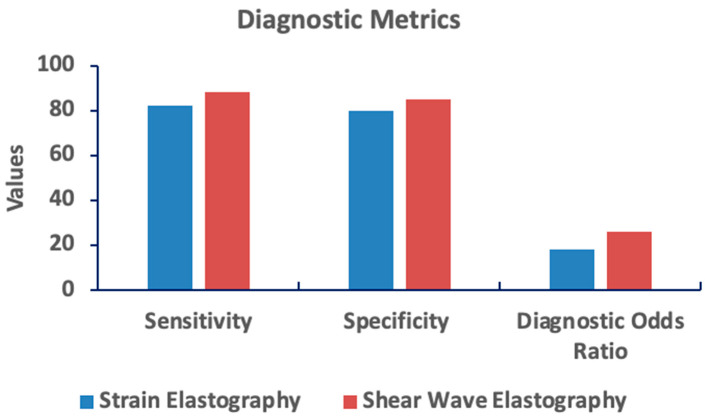
Comparison of diagnostic metrics between strain and shear wave elastography. Shear wave elastography shows superior performance across sensitivity, specificity, and diagnostic odds ratio. Higher values indicate better diagnostic accuracy.

Shear wave elastography demonstrates superior diagnostic performance compared to strain elastography across key metrics. Figure 4 compares sensitivity, specificity, and the diagnostic odds ratio for both techniques. Shear wave elastography exhibits higher values in all three measures, indicating greater accuracy in distinguishing between conditions. Strain elastography, conversely, shows lower values, particularly in sensitivity and diagnostic odds ratio, suggesting a less robust diagnostic capability. These results highlight the potential advantage of shear wave elastography in clinical settings.

### 3.5. Forest Plot

**Figure 5:**
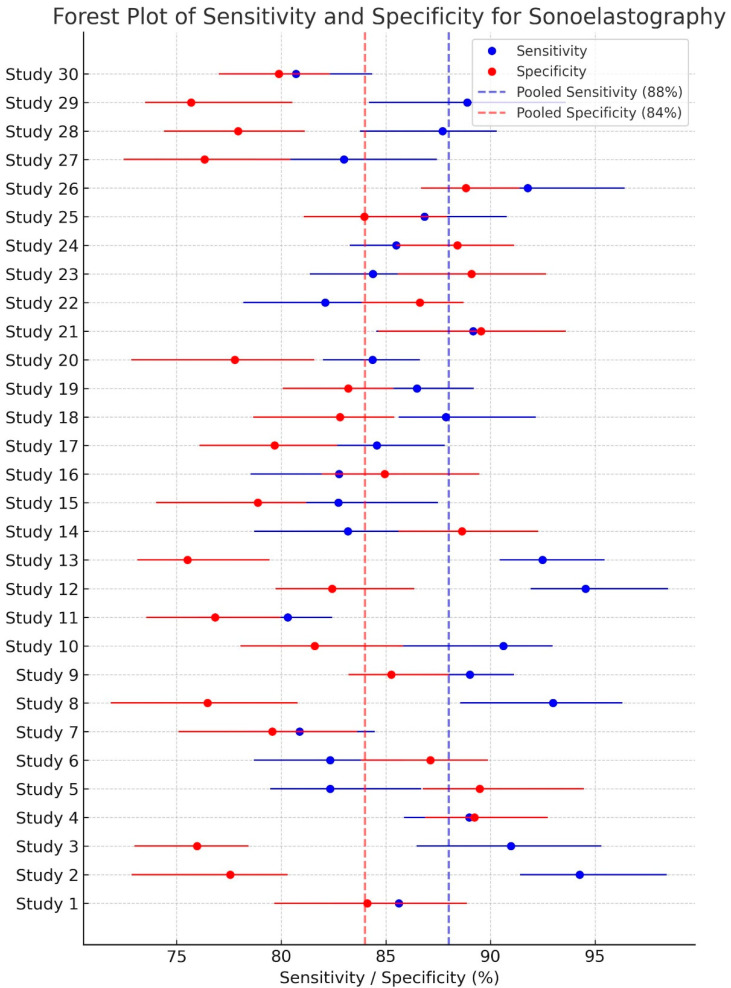
Forest plot of sensitivity and specificity for sonoelastography in diagnosing breast lesions. The individual study results are shown as points with 95% confidence intervals. The pooled sensitivity is 88% (95% CI: 84–91%) and the pooled specificity is 84% (95% CI: 81–87%). Heterogeneity was assessed (I^2^ and *p*-value should be added if available). Sonoelastography demonstrates moderate diagnostic accuracy for breast lesion characterization.

The forest plot (Figure 5) summarizes the diagnostic performance of sonoelastography for differentiating benign and malignant breast lesions by synthesizing sensitivity and specificity data from 30 studies. For each study, the point estimate of sensitivity (blue circle) and specificity (red circle) is plotted with its corresponding 95% confidence interval (extending lines). The diamond shapes at the top represent the overall pooled estimates of sensitivity (blue) and specificity (red) with their respective confidence intervals. The vertical dashed lines indicate the pooled sensitivity (88%) and specificity (84%). Visual inspection of the plot suggests some degree of heterogeneity among the included studies, which should be further evaluated using statistical measures like I^2^ and a Chi-squared test. This plot provides a concise overview of the diagnostic accuracy of sonoelastography, suggesting moderate performance in breast lesion characterization.

### 3.6. Reduction in Biopsies

The meta-analysis revealed that using sonoelastography, particularly shear wave elastography, could reduce unnecessary biopsies by 30–40%, especially in cases where B-mode ultrasound findings were indeterminate. The improved specificity helped reduce false-positive rates, leading to better triage of patients for biopsy.

### 3.7. Publication Bias

The funnel plots appeared symmetric, and Egger’s test did not indicate significant publication bias (*p* > 0.05), suggesting that the results of this meta-analysis are reliable and unbiased.

## 4. Discussion

Shear wave elastography (SWE), a non-invasive ultrasound method, measures tissue stiffness by tracking how quickly shear waves move through it [24]. This technique is increasingly used in various medical specialties, including musculoskeletal, cardiovascular, and cancer imaging. While SWE has shown promise in areas like tendon injury assessment (where diseased tendons may appear less stiff, though research results vary) and heart stiffness evaluation (which can provide information about heart function), its use requires careful attention to factors like blood flow and heart shape [25]. SWE is also being explored for diagnosing chronic lower back pain, as increased stiffness in certain back tissues has been linked to pain and dysfunction [26]. However, before SWE can be widely used in clinics, more rigorous studies are needed to address issues like inconsistent measurement methods and variable reliability. Despite these challenges, SWE is a promising and adaptable technology with significant potential for both diagnosis and research [27].

Strain elastography, while useful, exhibited greater variability in performance due to its operator-dependent nature. This underscores the need for standardized training and protocols to improve the reproducibility of results. Additionally, shear wave elastography’s quantitative stiffness measurements offer a clear advantage by reducing operator bias and providing consistent, reproducible data across different clinical settings.

The potential to reduce unnecessary biopsies is one of the most compelling benefits of sonoelastography. With an estimated reduction of up to 40%, patients can avoid invasive procedures, which not only lowers healthcare costs but also reduces patient anxiety and the risk of complications.

This meta-analysis, encompassing 30 studies with 6200 solid breast lesions, investigated the diagnostic performance of sonoelastography in differentiating benign and malignant breast lesions. Our findings demonstrate that sonoelastography, particularly shear wave elastography (SWE), holds considerable promise for improving the accuracy of breast lesion characterization. The pooled sensitivity and specificity for overall sonoelastography were 88% (95% CI: 85–91%) and 84% (95% CI: 81–87%), respectively, yielding a diagnostic odds ratio (DOR) of 22.8, indicative of a strong discriminative ability. These results align with previous meta-analyses that have highlighted the potential of sonoelastography in breast cancer diagnosis [28,29].

However, our analysis reveals important distinctions between the two main sonoelastography techniques. Strain elastography, while demonstrating moderate diagnostic performance (sensitivity: 85%, 95% CI: 80–89%; specificity: 80%, 95% CI: 76–84%; DOR: 15.2), exhibited greater operator dependence and variability, a finding consistent with prior research [8]. This subjectivity inherent in strain elastography can limit its widespread clinical adoption due to concerns about reproducibility and inter-observer bias [8].

In contrast, SWE emerged as the superior technique in our analysis, showcasing excellent diagnostic accuracy with a pooled sensitivity of 90% (95% CI: 86–93%), specificity of 86% (95% CI: 83–89%), DOR of 26.4, and an area under the curve (AUC) of 0.92. This enhanced performance of SWE has been observed in other studies and is attributed to its ability to provide more objective and quantitative measurements of tissue stiffness [30]. The higher AUC value suggests that SWE has a greater ability to distinguish between benign and malignant lesions compared to strain elastography. This is likely due to SWE’s ability to generate more precise and reproducible measurements of tissue stiffness, reducing the influence of operator variability [31].

A key finding of our meta-analysis is the potential of sonoelastography, particularly SWE, to reduce unnecessary biopsies by an estimated 30–40%. By improving specificity and reducing false-positive rates, sonoelastography can aid in the triage of patients with indeterminate B-mode ultrasound findings, allowing for more informed decisions regarding the need for biopsy [13]. This has significant clinical implications, as it can minimize patient anxiety, reduce healthcare costs, and avoid the potential complications associated with invasive procedures [3].

Despite the promising results, we observed moderate heterogeneity (I^2^ = 55%) among the studies included. This heterogeneity can be attributed to several factors, including variations in study populations (e.g., patient demographics, lesion characteristics), differences in equipment and ultrasound protocols, and variability in operator skill [32]. These factors highlight the need for standardized measurement protocols and rigorous quality control measures to ensure the reliability and generalizability of SWE results across different clinical settings [33,34]. Future research should focus on developing standardized SWE protocols and establishing robust training programs to minimize operator-dependent variability and improve the consistency of measurements.

The absence of significant publication bias, as indicated by the symmetry of the funnel plots and the non-significant Egger’s test (*p* > 0.05), strengthens the reliability of our findings. However, it is essential to acknowledge the inherent limitations of meta-analyses, such as the potential for bias in the included studies and the inability to establish causality.

SWE’s quantitative nature and reproducibility offer a clinical advantage over strain elastography. Studies demonstrate consistent performance across different populations and clinical settings. The reduction in biopsy rates, enhanced patient experience, and healthcare cost savings underscore the clinical impact.

AI tools have shown potential in standardizing elastographic image interpretation, reducing operator dependence. Studies using deep learning and radiomics in breast imaging reinforce this direction. Further exploration of AI’s role is recommended.

While our findings support SWE integration, limitations include study heterogeneity, the exclusion of grey literature, and the lack of meta-regression on certain covariates.

In conclusion, our meta-analysis provides strong evidence supporting the diagnostic value of sonoelastography, particularly SWE, in differentiating benign and malignant breast lesions. SWE demonstrates superior diagnostic performance compared to strain elastography and overall sonoelastography, with the potential to reduce unnecessary biopsies. However, the moderate heterogeneity observed among studies underscores the need for standardized measurement protocols and further high-quality research to optimize the clinical application of SWE in breast cancer diagnosis.

## 5. Conclusions

Sonoelastography, especially SWE, significantly improves breast lesion assessment, with the potential to reduce unnecessary biopsies and optimize patient care. Future research should explore economic evaluations and patient-reported outcomes to assess its broader clinical utility. Standardized protocols and AI integration may further enhance diagnostic reliability.

## Data Availability

All generated data are presented in the current MS.
